# OpenDMAP: An open source, ontology-driven concept analysis engine, with applications to capturing knowledge regarding protein transport, protein interactions and cell-type-specific gene expression

**DOI:** 10.1186/1471-2105-9-78

**Published:** 2008-01-31

**Authors:** Lawrence Hunter, Zhiyong Lu, James Firby, William A Baumgartner, Helen L Johnson, Philip V Ogren, K Bretonnel Cohen

**Affiliations:** 1 Center for Computational Pharmacology, University of Colorado School of Medicine, Aurora, CO 80045, USA; 2 National Center for Biotechnology Information, National Library of Medicine, Bethesda, MD 20894, USA; 3 PowerSet, Inc., San Francisco, CA 94107, USA; 4 Department of Computer Science, University of Colorado, Boulder, CO 80303, USA

## Abstract

**Background:**

Information extraction (IE) efforts are widely acknowledged to be important in harnessing the rapid advance of biomedical knowledge, particularly in areas where important factual information is published in a diverse literature. Here we report on the design, implementation and several evaluations of OpenDMAP, an ontology-driven, integrated concept analysis system. It significantly advances the state of the art in information extraction by leveraging knowledge in ontological resources, integrating diverse text processing applications, and using an expanded pattern language that allows the mixing of syntactic and semantic elements and variable ordering.

**Results:**

OpenDMAP information extraction systems were produced for extracting protein transport assertions (transport), protein-protein interaction assertions (interaction) and assertions that a gene is expressed in a cell type (expression). Evaluations were performed on each system, resulting in F-scores ranging from .26 – .72 (precision .39 – .85, recall .16 – .85). Additionally, each of these systems was run over all abstracts in MEDLINE, producing a total of 72,460 transport instances, 265,795 interaction instances and 176,153 expression instances.

**Conclusion:**

OpenDMAP advances the performance standards for extracting protein-protein interaction predications from the full texts of biomedical research articles. Furthermore, this level of performance appears to generalize to other information extraction tasks, including extracting information about predicates of more than two arguments. The output of the information extraction system is always constructed from elements of an ontology, ensuring that the knowledge representation is grounded with respect to a carefully constructed model of reality. The results of these efforts can be used to increase the efficiency of manual curation efforts and to provide additional features in systems that integrate multiple sources for information extraction. The open source OpenDMAP code library is freely available at

## Background

Conceptual analysis is the process of mapping from natural language texts to a formal representation of the objects and predicates (together, the concepts) meant by the text. The history of attempts to build programs to do conceptual analysis dates back to at least 1967 [1]. Recent advances in the availability of high quality ontologies, in the ability to accurately recognize named entities in texts, and in language processing methods generally have made possible a significant advance in concept analysis, arguably the most difficult and general natural language processing task. Here we report on the design, implementation and several evaluations of OpenDMAP, an ontology-driven, integrated concept analysis system that significantly advances the state of the art. We also discuss its application to three important information extraction tasks in molecular biology.

Information extraction (IE) efforts are widely acknowledged to be important in harnessing the rapid advance of biomedical knowledge, particularly in areas where important factual information is published in a diverse literature. In a recent *PLoS Biology *essay Rebholz-Schuhmann [2] argued, "It is only a matter of time and effort before we are able to extract facts [from articles in the primary literature] automatically. The consequences are likely to be profound." Existing examples include extraction of information about gene-gene interactions [3], alternative splicing [4], functional analysis of mutations [5], phosphorylation sites [6], and regulatory sites [7]. The primary significance of OpenDMAP to these efforts is that it leverages the large-scale efforts being made in biomedical ontology development, such as the Open Biomedical Ontologies Foundry (OBO Foundry) [8].

Logical representations of reality, such as those built on the OBO Foundry, use a set of predicates that formally describe properties of, or relationships among, objects. Predicates are defined with a specific number and type of admissible arguments. For example, the predicate *expresses *might be specified to take two arguments, a gene and a cell type, meaning that the specified gene is expressed in all normal cells of the specified type. Such predicates can also be related to each other through abstraction ("is a") and packaging ("part of") hierarchies, as done in the OBO Foundry. The semantics defined by the predicates and hierarchies in such ontologies provide a powerful tool for natural language processing.

Independently constructed ontologies have played at best a modest role in prior natural language processing systems. Guarino [9] characterizes various uses of ontologies in information systems: only systems that use an ontology at run time (rather than during system construction) to explicitly represent the domain knowledge exploited by the system qualified for what Guarino called an "ontology-driven information system proper." To our knowledge, OpenDMAP is the first system developed to exploit a community consensus ontology as the central organizing principle of an information extraction system; for example, none of the systems that participated in the 2004 TREC Genomics evaluation for recognizing instances of Gene Ontology terms in text [10] meet the Guarino definition. Other language processing systems have used either small, *ad hoc *conceptual representations developed specifically for the application, or structured linguistic resources, such as WordNet [11], which do not meet the logical requirements for an ontology. While the implementation reported below exploits only a small portion of the OBO Foundry, and the crucial Relationship Ontology component of the Foundry is still in an early stage of development, the organizing principles of OpenDMAP generalize straightforwardly.

The MetaMap system [12] identifies biomedical concepts from free-form textual inputs and maps them to entries in the Unified Medical Language System (UMLS) metathesaurus; SemRep [13] is a related system that maps to predications drawn from the UMLS semantic network, and SemGen [14,15] is another related system that is focused on mapping to UMLS terms relevant to the etiology of genetic disease. These systems and their extensions have been used to extract semantic relationships relevant to pharmacogenomics [16] and to compare alternative sources of information [17], among other applications. OpenDMAP is like MetaMap and its descendents in that it can only produce output drawn from a predefined semantic representation. The main difference is that MetaMap, SemRep and SemGen are structured as traditional NLP systems, with a lexicon that enumerates possible concepts that might be associated with a word or phrase. Multiple possible mappings are returned, with rankings. OpenDMAP provides an alternative method of organizing knowledge about language, so that each concept has associated with it a set of patterns that describe how that concept can be realized in language; there is no explicit lexicon.

To appreciate the differences between OpenDMAP and previous work in biomedical text mining, it is also useful to contrast its handling of syntactic structure and of semantic content with other systems. At one end of the spectrum are systems that employ essentially asyntactic representations. Early in the modern period of genomic natural language processing, some such systems were able to achieve significant (and in some cases ground-breaking) results using techniques based on text literals only. These include [18-20]. One line of subsequent work has attempted to increase the coverage of these early systems, which utilized manually-built patterns, by automatically acquiring considerably larger sets of patterns – see, for example, Huang et al. 2004 [21]. Another line of subsequent work has focused on adding a modest, but still useful, level of linguistic abstraction by explicitly including either lexical categories (parts of speech), word stems, or both [22,23]. These systems were essentially agrammatical; in contrast, OpenDMAP utilizes a classic form of "semantic grammar," freely mixing text literals, semantically typed basal syntactic constituents, and semantically defined classes of entities.

Although OpenDMAP is capable of utilizing full syntactic parses, the patterns for the three separate tasks discussed in this paper utilize primarily shallow syntactic parses (the development phase of the transport project reports results using syntactic dependency information). It remains to be seen what depth of syntactic parsing is useful in biomedical text mining. Some early systems explored full parsing [24,25], but they were not generally fruitful, and typical systems have employed at most shallow parsing [26-28]; only recently has productive attention returned to syntactically ambitious approaches to biomedical text [29-31], much of it taking a dependency-based, rather than a constituent-based, approach.

All of the systems discussed thus far have in common the fact that they employ some notion of explicit patterns, be they agrammatical, syntactic, or semantic. In a separate line of work, patterns are entirely implicit – that is, they exist only to the extent that they are captured by orthogonal features. This work approaches relation extraction as a classification problem; a classic example is the work of Craven and Kumlein 1999 [32]. Bunescu et al. 2005 [33] presents a detailed analysis of a number of classification-based approaches; the state of the art is characterized by the participants in the recent BioCreative protein-protein interaction shared task [34].

OpenDMAP has been applied in three domains: protein transport, protein-protein interaction and the expression of a gene in a particular cell type. The three application domains are independently significant. Protein transport, the directed movement of proteins from one cellular compartment to another, is a broadly important biological phenomenon. Although protein subcellular localization information is centralized (e.g. through ontological annotations at NCBI and in various model organism databases), information about transport is not. Protein transport information is published throughout the scientific literature, but no previous method was able to capture it systematically. Protein-protein interaction extraction has been the subject of dozens of systems (see, e.g. a review in [35]). Widely used web resources such as IHOP [3] and Chilibot [36] are based entirely on automated extraction of protein-protein interactions from text. This task was used in the BioCreative community evaluation, described below. The third application area, extraction of assertions that a particular gene is expressed in a particular cell type, is of significance since it appears to be the predicate found most frequently in the biomedical literature; a form of the verb "express," usually its nominalization "expression," appears in nearly 20% of NCBI's GeneRIFs [37].

The protein transport task is illustrative of another distinguishing aspect of the OpenDMAP approach: it provides mechanisms for handling relationships involving more than two entities. Note that the protein transport predicate has at least three arguments: what protein is transported, from where, and to where (our model also includes a fourth argument: the transporting protein). Although some linguistic expressions of the concept may elide an argument, the predicate itself inherently describes a greater than binary relationship. Wattarujeekrit et al. [38] and Cohen and Hunter [39] present evidence that many important predicates in biomedicine require more than two arguments. However, most previous efforts at extracting relationships from biomedical text have addressed exclusively binary relationships. Geneways [40] and RLMPS-P [41] are the only other biomedical IE systems of which we are aware that extracted greater than binary relationships, and neither is ontology-driven.

Assessing the accuracy of an information extraction system is a very labor-intensive activity. In order to identify information that could have been extracted, but was not (a "false negative"), a person must go through a large volume of text to determine all of the relevant assertions. To estimate the reliability of these manually derived assertions, at least two people must complete that task to assess inter-rater reliability. Once such data is used for one evaluation and system developers have seen it, further use of the data will generate upwardly biased accuracy estimates as system developers fit their systems to it. For these reasons, large-scale community evaluations of information extraction systems are particularly important. The second Critical Assessment of Information Extraction in Biology, (BioCreative) [34,42], community evaluation included a test of systems designed to extract human protein-protein interaction information from the full texts of hundreds of journal articles, called the IPS task. Human curators from the IntAct database [43] manually extracted interaction assertions from these articles using the same curatorial standards as for the database. The results produced by human experts were compared to the results submitted from 45 systems developed by laboratories around the world, providing the best current assessment of the accuracy of protein interaction information extraction systems. The performance of OpenDMAP on the protein interaction task was evaluated as part of this shared task. More limited evaluations of the accuracy in the other applications are also reported in the results section.

The accuracy of an information extraction system depends on the genre of texts on which it operates [44]. This report demonstrates the application of OpenDMAP to full texts of scientific journal articles, to Medline abstracts, and to GeneRIFs (single sentences or sentence fragments that are selected by human curators for relevance to the function of a particular gene product). GeneRIFs are particularly attractive targets for information extraction, due to their roughly sentential length (identified by [44] as the optimum), breadth of coverage, manual preselection for relevance, and association with at least one normalized gene reference. Despite these attractive features, this is the first report of an information extraction system targeting them.

## Results

OpenDMAP information extraction systems were produced for extracting protein transport assertions (transport), protein-protein interaction assertions (interaction) and assertions that a gene is expressed in a cell type (expression). Each of these systems was run over all abstracts in Medline as of June 18, 2007, producing a total of 72,460 transport instances, 265,795 interaction instances and 176,153 expression instances. These results are provided in RDF format in the Additional Files 1, 2, 3, 4.

**Figure 1:**
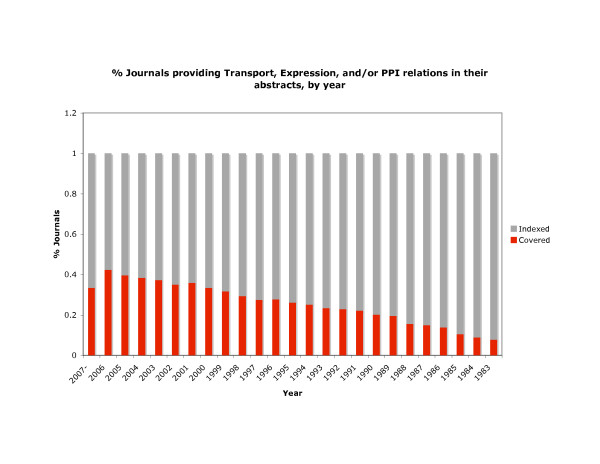
**OpenDMAP coverage of MEDLINE**. The gray bars indicate the number of journals indexed by MEDLINE each year. The red bars indicate the number of journal abstracts from which OpenDMAP extracted at least one assertion regarding transport, interaction or expression. In recent years, more than 40% of biomedical journals contain such information. 2007 is partial data (through July 1).

One particularly striking result is the diversity of journals from which these assertions were mined. The transport relationships were extracted from 2,340 different journals; the interaction relationships from 4,103 different journals; and the expression relationships from 2,984 different journals. A total of 4,434 unique journals contributed to these results, nearly 40% of the journals indexed in Medline each year (see Figure 1).

For the BioCreative evaluation, the interaction system was run on the full texts of all of the 359 articles in the test set, producing 385 interaction assertions. Performance was averaged per article, since a few articles had a very large number of interactions and would have dominated a per assertion calculation. OpenDMAP's average F-measure of 0.29 was 10% higher than the next best scoring system, and more than three standard deviations above the mean performance. OpenDMAP's recall was similar to the other high scoring systems; its advantage arose from being substantially more precise (fewer false positives), achieving an average precision of 0.39, more than 20% better than the next best system. Due to IntAct's curation criteria, which require clear experimental evidence for an interaction in the text, these results are quite conservative. Many "false positives" were in fact assertions of interactions, but fell short of the evidential requirements for IntAct curation.

A manual evaluation of the performance of the protein transport recognition system was based on all 570 GeneRIFs containing a form of the word "translocate" (382 of which were about protein transport, and 188 were about the transport of something else). Since transport is a greater than binary relationship, the extraction was only counted as correct if all of the components extracted matched the human annotation. For that strict criterion, OpenDMAP achieved precision of 0.75 and a recall of 0.49 (F-score of 0.59). If incomplete extractions are counted as correct, precision is unchanged at 0.75 and recall rises to 0.67 (F-score of 0.71). A substantial proportion of the errors were due to imperfect recognition of proteins; if OpenDMAP is given correct protein identifications as inputs, precision is 0.77, strict recall is 0.67 (F-score of 0.72) and incomplete recall is 0.85 (F-score of 0.81).

A manual evaluation of the performance of the expression recognition system was based on 324 GeneRIFs containing a form of the word "express," (these sentences contained 469 assertions about expression, 205 of which were about gene expression in 178 different cell types). Open DMAP had a precision of 0.64, but missed many statements that annotators identified as expression assertions, achieving a recall of only 0.16 (F-score of 0.26). A substantial portion of these errors were due to imperfect recognition of gene names; if OpenDMAP is given correct gene identifications as input, precision is 0.85 and recall is 0.36 (F-score of 0.51). Many other failures to identify expression assertions were related to coordination; the test set had an average of more than two expression assertions per sentence, but the IE system extracted only about 1.3 assertions per sentence.

## Discussion

As demonstrated by its performance in the community evaluation, OpenDMAP advances the state of the art for extracting protein-protein interaction predications from the full texts of biomedical research articles. Furthermore, this level of performance appears to generalize to other information extraction tasks, including extracting information about predicates of more than two arguments.

There are several reasons why OpenDMAP exhibits better performance than any other biomedical information extraction system to date. OpenDMAP is an extension of the Direct Memory Access Parsing (DMAP) paradigm described in [45] and [46]. Three innovations distinguish the present work from those prior efforts. First, the ontology component of OpenDMAP is independent of the rest of the system. The knowledge representation component is the well-established, open source Protégé ontology development system [47,48], and OpenDMAP concept analyzers can be associated with any ontology compatible with Protégé, for example, the OBO Foundry. Second, OpenDMAP is fully integrated with the open source Unstructured Information Management Architecture, (UIMA) [49-51], which allows the results of any text processing application interfaced to UIMA to be exploited by the OpenDMAP system. As demonstrated below, this mechanism facilitates the use of many external language processing systems, including tokenizers, sentence boundary detectors, entity recognition systems, and syntactic parsers. Since the inputs and outputs of each system are mapped by UIMA to a common annotation structure accessed by OpenDMAP, the use, comparison and combination of various approaches to language processing can all be fully integrated into OpenDMAP patterns. The third innovation in the OpenDMAP system is an expanded pattern language for specifying how concepts can be expressed in text. The pattern language not only allows specifications of mixtures of any concepts available from either the ontology (e.g. a protein transport process) or the results of UIMA text processing (e.g. the head of a noun phrase), but it also has new features that allow more flexible concept ordering than previous DMAP analyzers (see the description of the pattern language in the Methods section for details).

The intimate connection between the ontology and the natural language processing system provides two significant advantages over prior information extraction systems generally. First, the output of the information extraction system is always constructed from elements of the ontology, ensuring that the knowledge representation is grounded with respect to a carefully constructed model of reality. In contrast, the outputs of most natural language processing systems are grounded only in substrings of text, not normalized to any model at all. Progress in normalizing biological entities recognized in text to specific database identifiers [52-54] has made the output of text processing systems much more valuable. Mapping the properties and relationships extracted to a community ontology similarly provides a significant increment in the value of the output from text processing systems.

The second advantage of the OpenDMAP approach is that all of the knowledge used by the system to recognize concepts is structured by the ontology. In contrast, the nearly universal alternative approach is to embody knowledge of language into a lexicon, which associates individual lexical items with their possible semantic interpretations. In the OpenDMAP approach, information about which concepts are potentially relevant to the analysis of a particular text passage straightforwardly places limits on the linguistic knowledge relevant to analyzing that passage. This approach finesses many difficult ambiguity resolution problems faced by lexicon-driven systems, since these limits on the knowledge applied to conceptual analysis prevent many multiple interpretation problems from arising at all. For example, the string "hunk" refers to a cell type (human natural killer cells), a gene (hormonally upregulated Neu-associated kinase), and the general English word meaning a large piece of something without definite shape. A traditional, lexicon-driven system would have an explicit method for assigning the correct word sense to any occurrence of the string "hunk." However, OpenDMAP patterns specify expectations of semantic classes (e.g. in the transport application described below, the transported entity must be a protein or a molecular complex); if it is possible to construe a string as an instance of an expected class, the pattern matches. The fact that there might be possible alternative interpretations of the matching string has no consequence, and no explicit ambiguity resolution step is necessary. Ambiguity is a leading cause of errors in text processing systems, and this approach is one of the contributing factors to OpenDMAP's superior performance. Our top-down approach to restricting possible interpretations does not address all problems due to ambiguity in language; for example, errors in preprocessing systems (e.g. syntactic parsing, see below) are not effected.

The use of UIMA greatly facilitates the incorporation of various applications as input to OpenDMAP. The outputs of NLP tools integrated into the system are described by the extensible UIMA type system. In the case that a new type of information is produced by a preprocessor, OpenDMAP patterns would have to be modified to take advantage of the new type of information available. For example, the first time an external cell type tagging system is added, the UIMA type of the result of that processor must be linked to a cell-type concept in an OpenDMAP ontology in order for it to be used in patterns. However, if a new NLP tool produces a UIMA output type that has been used by OpenDMAP previously, then no changes in the ontology or patterns are needed.

We believe that the outputs of information extraction systems are not likely to useful until the F-score (or at least the precision) is greater than about 0.85 [34], so the various sources of error in these systems must be addressed. A significant cause of errors in the OpenDMAP system as evaluated is incorrect identification of gene and protein names. The UIMA architecture makes it trivial to adopt and exploit better gene/protein recognition systems as they are developed. The best gene name identification and normalization systems from the BioCreative assessment achieved F-scores greater than 0.8, significantly above the ~0.7 F-score of the ABNER system [55] used by OpenDMAP to achieve the reported performance. Use of such a system should improve the performance of OpenDMAP.

Error analysis of the false positives in the transport data set indicates that more than 80% are due to errors in the syntactic analysis. For example, in the sentence "Rho protein regulates the tyrosine phosphorylation of FAK through translocation from the nucleus to the membrane," the subject of the translocation was incorrectly identified as FAK (rather than Rho) by the Stanford parser. That parser was developed for general English rather than biomedical text, so using specialized syntactic analysis systems may improve the precision of OpenDMAP. Remaining problems in false positives are due to problematic tokenization, failures to properly resolve anaphoric reference, and, rarely, negation. False negatives are due to gaps in concept recognition patterns, more than half of which arise from a failure to properly handle coordinated clauses and conjunctions. Addressing these issues remains an open area of research.

Another issue was that the Stanford parser was too slow to use in the application of the transport system to all of Medline, so it wasn't run. OpenDMAP ignores aspects of patterns that require inputs that aren't present, so the patterns that contained syntactic dependencies did not have to be altered. These syntactic constraints are important for accuracy, however. Tested on the gold standard set for the system without the parser precision drops to 0.62, while strict recall remains largely unchanged, rising to 0.51.

## Conclusion

Despite OpenDMAP elevating the state of the art for biomedical information extraction significantly beyond previous levels, error rates remain high. In the most challenging BioCreative task, finding curatable assertions in full text documents, only about 29% of the relevant assertions were found, and only about 39% of the extracted assertions were completely correct. Such error rates mean that automatically generated databases cannot replace manual curation efforts. However, the evidence is quite clear that manual curation cannot keep up with the rate of data generation [56]. The surprisingly large number of journals that contained information relevant to these three IE tasks suggests that the temporal approach taken in [56] may actually underestimate the severity of the problem.

Although the outputs produced by large-scale IE systems are not yet suitable for producing factual databases for direct use by biomedical researchers, the current level of performance provides two important facilities to the research community. First, the results of these efforts can be used to significantly increase the efficiency of manual curation efforts. Each extracted assertion is tied to a specific text, which can be used to direct the attention of manual curators both to relevant documents and to specific relevant passages within a document. Effective integration of IE results into curatorial workflows will require the development of new tools. OpenDMAP developers are working with curators at IntAct to address these issues. The open source availability of OpenDMAP will facilitate the work of others addressing this issue as well. The second important use of the sorts of results that IE systems are currently able to generate is in statistical integration with multiple sources of noisy data, such as those described in [57] and [58]. As demonstrated in the latter, the proper addition of even noisy data from the literature substantially improves the quality and coverage of protein-protein interaction networks for several species.

## Methods

**Figure 2:**
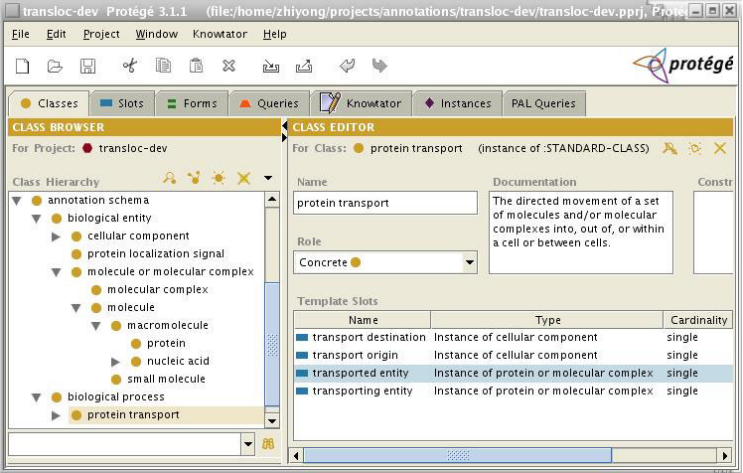
**Screenshot of the Protégé ontology for the protein transport task**. The slots of the protein transport class are shown in the lower right panel of this screen shot. Note that the subclasses of Cellular Component and Protein Transport are not shown.

OpenDMAP uses Protégé [47] to provide an object model for the possible concepts (predicates and objects) that might be found in a text. Protégé models concepts (including actions) as classes that participate in abstraction and packaging hierarchies, and relationships as class-specific slots. For example, protein transport is modeled as a class (called PROTEIN-TRANSPORT) and the relationship between a transport event and the protein transported in that event is represented as a slot in that class (called [TRANSPORTED-ENTITY]). Slots can take on values, which can be constrained to be instances of other classes. For example, the [TRANSPORTED-ENTITY] slot of the PROTEIN-TRANSPORT class is constrained to be an instance of either of the classes PROTEIN or MOLECULAR-COMPLEX. Figure 2 shows a portion of the model used for the transport, which includes biological entities, such as molecular complexes and cellular components, and biological processes, particularly protein transport. This model is drawn almost entirely from the Gene Ontology (GO) [53] although the relationships that define the four slots shown in Figure 2 are from a provisional submission to the OBO Foundry Relationship Ontology and are not official. Preprocessing tools (ABNER [55] and LingPipe [59]) were applied to tag instances of proteins, genes, and cell types.

For the transport task, patterns were produced for 30 ontology concepts; eight directly related to transport and 22 others for cellular components that are the sources and destinations of transport. A large number of other concepts (e.g. genes, proteins and cell types) do not have explicit patterns associated with them, but are instead tagged as such by UIMA tools during preprocessing. The protein-protein interaction task involved producing patterns for nine concepts, and the cell expression task required patterns for six additional concepts.

The UIMA architecture [49] manages the processing of document sets. The collection of document processing tools interfaced through UIMA includes the LingPipe tools for sentence boundary detection and tokenization [59], both the LingPipe and ABNER [55] tools for recognizing protein mentions, ABNER for recognizing mentions of cell types, the Stanford Parser [60] to provide syntactic trees, and a locally produced implementation of the Modified Hobbs algorithm [61] for anaphora resolution. Various combinations of these tools were used in the different applications. For example, the GeneRIFs used in the transport application did not require sentence segmentation, and the applications to all medline abstracts did not use syntactic elements in patterns because the Stanford Parser was too slow to run over all of Medline. The results of this preprocessing are stored in UIMA's common annotation structure.

In order to be able to recognize a concept in text, OpenDMAP associates one or more patterns with each concept. A pattern describes the words, phrases, parts of speech, syntactic structures or concepts that should cause an instance of the associated concept to be recognized. A simple pattern, such as the one shown in equation 1, enumerates a disjunction of words that should trigger recognition of a concept.

(1) NUCLEUS := nucleus, nuclei, nuclear;

The patterns for all of the CELLULAR-COMPONENT concepts were derived from the GO term names and synonyms, supplemented with derivational variants, such as the adjectival "nuclear" in equation 1. Twenty-two GO cellular component terms were used, along with 19 synonyms associated with the GO terms, and 78 additional derivational variants generated by inspection of the training corpus.

More complex patterns can include references to non-terminals, particularly other concepts. Equation 2 is one of the patterns for recognizing instances of the PROTEIN-TRANSPORT concept. This pattern specifies that a reference to PROTEIN-TRANSPORT can appear in text as a reference to a concept that can fill the [TRANSPORTED-ENTITY] slot, followed by the word "translocation" followed optionally by a phrase beginning with the word "from", possibly including a word with the part of speech determiner, and a concept that could fill the [TRANSPORT-ORIGIN] slot, also followed optionally by a similar phrase that regarding the [TRANSPORT-DESTINATION] slot.

(2) $$\begin{array}{c} PROTEIN-TRANSPORT\text{ :=}\left[TRANSPORTED-ENTITY\right]translocation\\ \left(from\left\{\det\right\}?\left[TRANSPORT-ORIGIN\right]\right)\text{?}\\ \left(to\left\{\det\right\}?\left[TRANSPORT-DESTINATION\right]\right)\text{?;} \end{array}$$

When a pattern that includes a slot name is matched, the instance created has its slots filled with the concepts that matched the slot names in the pattern. For example, the above pattern matches the GeneRIF that contains "... Bax translocation to mitochondia..." (from Entrez GeneID 27113). Bax, which is recognized as a protein by ABNER, will cause an instance of the protein concept to be created; an instance of a protein matches one of the constraints on filler of the [TRANSPORTED-ENTITY] slot, which causes that slot to match to the Bax protein concept. The word "translocation" matches, and, while the optional "from" clause does not match, the "to" clause does match, since "mitochondria" matches one of the patterns for a subclass of CELLULAR-COMPONENT, the constraint on the filler of the [TRANSPORT-DESTINATION]. Since the entire pattern matches, an instance of PROTEIN-TRANSPORT is created, with the Bax protein concept in its [TRANSPORTED-ENTITY] slot and an instance of the mitochondria concept (from GO's cellular component hierarchy) in its [TRANSPORT-DESTINATION] slot.

OpenDMAP patterns can express variability in word and phrase order. Note, for example, that equation 2 would fail to match the phrase "Bax translocation to mitochondria from the cytosol." The special pattern marker @ is used to identify a set of subpatterns that are both optional and can occur before or after a required phrase; multiple @ marked phrases can occur in any order. For example, equation 2 can be modified with this marker to recognize the above text:

(3) $$\begin{array}{c} \text{PROTEIN-TRANSPORT := ([TRANSPORTED-ENTITY] translocation)}\\ \text{@ (from \{det\}? [TRANSPORT-ORIGIN])}\\ \text{@ (to \{det\}? [TRANSPORT-DESTINATION]);} \end{array}$$

Many sentences in the literature express multiple concepts, making extraction of even simple assertions problematic. Consider the following GeneRIF from GeneID:29560: "...HIF-1alpha which is present in glomus cells translocates to the nucleus...." The intervening phrase "which is present in glomus cells" prevents the pattern in equation 3 from matching that sentence. OpenDMAP does have a wildcard character (underscore) that could be added to the pattern in equation 3, between the [TRANSPORTED-ENTITY] concept and the word "translocation," allowing this sentence to be matched. However, using such a wild card would make any protein mentioned before the word "translocation" match the pattern, which is too promiscuous. To address this problem, OpenDMAP allows patterns to specify syntactic constraints on potential matches. For example, the [TRANSPORTED-ENTITY] slot can be constrained to have a syntactic dependency on the head of a phrase that contains the translocate action, thereby constraining it both semantically (it must be a protein or molecular complex) and syntactically (it must be the subject, object or modifier of the translocation). Furthermore, the reliance on the exact word "translocation" can be relaxed to be any reference to a transport action word, including both verbal and nominal forms of multiple terms (e.g., transported, translocation). The PROTEIN-TRANSPORT class is extended to have an [action] slot that specifies the type of transportation action, to keep track of the term that was used. Equation 4 demonstrates the pattern language for specifying syntactic constraints:

(4) $$\begin{array}{l} \text{PROTEIN-TRANSPORT := ([TRANSPORTED-ENTITY dep:x] \_}\\ \text{[action ACTION-TRANSPORT head:x])}\\ \;\text{@ (from \{det\}? [TRANSPORT-ORIGIN])}\\ \;\text{@ (to \{det\}? [TRANSPORT-DESTINATION]);} \end{array}$$

The use of the variable "x" in the specification identifies a specific syntactic unit, linking the dependency to the head of a phrase. Multiple variables can be used to specify constraints on different syntactic units within a sentence.

OpenDMAP patterns are very powerful. Only five such patterns, shown in equations 5–9 were required for the transport extraction system performance noted above. These patterns were devised manually, based on expert knowledge of the domain and on a small training set of sample GeneRIFs.

(5) $$\begin{array}{c} \text{PROTEIN-TRANSPORT := [TRANSPORT-DESTINATION] [action ACTION-TRANSPORT] \_}\\ \text{(of \{det\}? [TRANSPORTED-ENTITY])?}\\ \text{(by \{det\}? [TRANSPORTING-ENTITY])?;} \end{array}$$

(6) $$\begin{array}{l} \text{PROTEIN-TRANSPORT := ([TRANSPORTED-ENTITY dep:x] \_}\\ \text{[TRANSPORT-DESTINATION]}\\ \text{[action ACTION-TRANSPORT head:x])}\\ \text{(by \{det\}? [TRANSPORTING-ENTITY])?;} \end{array}$$

(7) $$\begin{array}{l} \text{PROTEIN-TRANSPORT := [action ACTION-TRANSPORT]}\\ \text{@ (of \{det\}? [TRANSPORTED-ENTITY])}\\ \text{@ (by \{det\}? [TRANSPORTING-ENTITY])}\\ \text{@ (from \{det\}? [TRANSPORT-ORIGIN])}\\ \;\text{@ (to|toward|towards|into \{det\}?}\\ \text{[TRANSPORT-DESTINATION]);} \end{array}$$

(8) $$\begin{array}{l} \text{PROTEIN-TRANSPORT := ([TRANSPORTED-ENTITY dep:x] \_}\\ \text{[action ACTION-TRANSPORT head:x])}\\ \text{@ (by \{det\}? [TRANSPORTING-ENTITY])}\\ \text{@ (from \{det\}? [TRANSPORT-ORIGIN])}\\ \text{@ (to|toward|towards|into \{det\}?}\\ \text{[TRANSPORT-DESTINATION]);} \end{array}$$

(9) $$\begin{array}{l} \text{PROTEIN-TRANSPORT := ([TRANSPORTED-ENTITY] (is|were|are|was)}\\ \text{[action ACTION-TRANSPORT-PASSIVE])}\\ \text{@ (by \{det\}? [TRANSPORTING-ENTITY])}\\ \text{@ (from \{det\}? [TRANSPORT-ORIGIN])}\\ \text{@ (to|toward|towards|into \{det\}?}\\ \text{[TRANSPORT-DESTINATION]);} \end{array}$$

These patterns were augmented with 119 cellular component patterns.

The test data used in the transport and expression evaluations were marked up by domain experts trained in conceptual annotation, using the Knowtator annotation tool [62].

## Availability of data and software

The OpenDMAP platform-independent Java 1.5 source code, including UIMA wrappers for the tools used in this work and the patterns for the three tasks, is available from  under the Mozilla Public License v1.1 (OpenDMAP) and GPL v2.0 license (UIMA wrappers). The results of the information extraction effort are available as RDF format files in the Additional Files 1, 2, 3, 4.

## Authors' contributions

LH conceived of the project, supervised the design and implementation of the system and wrote the manuscript. ZL was responsible for the transport project, including writing the patterns and analyzing the results; he also contributed suggestions for the interaction task. JRF implemented the OpenDMAP pattern recognition engine and its UIMA wrapper. WAB wrote all other infrastructure software, including the other UIMA wrappers, managed the data, applied OpenDMAP to all of MEDLINE, and designed and built other software. HLJ was responsible for the interaction and expression projects, including writing the patterns, analyzing the results, and doing the associated error analyses. PVO managed the creation of the gold standard data for transport and contributed to the design of the pattern language syntax. KBC managed the team, selected the preprocessing tools, coordinated and supervised the interaction and expression task efforts, and provided linguistic and software design contributions during all phases of the project. All authors have read and approved this manuscript.

## Supplementary Material

Additional file 1 **Transport instances from MEDLINE**. This file contains the RDF formatted instances of *transport*, mined from MEDLINE with OpenDMAP. Click here for file

Additional file 2 **Interaction instances from MEDLINE, part 1**. The *interaction *data set is very large. This file contains the first half of RDF formatted instances of *interaction*, mined from MEDLINE with OpenDMAP. Click here for file

Additional file 3 **Interaction instances from MEDLINE, part 2**. The *interaction *data set is very large. This file contains the second half of RDF formatted instances of *interaction*, mined from MEDLINE with OpenDMAP. Click here for file

Additional file 4 **Expression instances from MEDLINE**. This file contains the RDF formatted instances of *expression*, mined from MEDLINE with OpenDMAP. Click here for file
